# ‘Mum never loved me.’ How structural factors influence adolescent sexual and reproductive health through parent–child connectedness: A qualitative study in rural Tanzania

**DOI:** 10.2989/16085906.2014.945387

**Published:** 2014-07-21

**Authors:** Joyce Wamoyi, Daniel Wight

**Affiliations:** ^a^ National Institute for Medical Research, Department of Sexual and Reproductive Health, PO Box 1462, Mwanza, Tanzania; ^b^ Medical Research Council, Department of Social and Public Health Sciences Unit, 4 Lilybank Gardens, Glasgow, G12 8RZ, UK

**Keywords:** ASRH, parent–child connectedness, parenting, structural factors, sub-Saharan Africa, Tanzania

## Abstract

Research in high income countries shows parent–child connectedness to be protective against undesirable sexual and reproductive health (SRH) outcomes among young people. Little has been done to understand the nature of parent–child connectedness, the structural factors that impact on connectedness and parents’ understanding of how connectedness affects their children's sexual behaviour in sub-Saharan Africa and Tanzania in particular. Ethnographic research involved 30 days of observation in 10 households, 9 focus group discussions and 60 in-depth interviews. Thematic analysis was conducted using NVIVO qualitative data analysis software.

The structural factors with greatest influence on connectedness were economic circumstances, gender, social status, state education, and globalisation. Economic circumstances impacted on parent–child connectedness through parents’ ability to provide for their children's material needs, and the time their occupation allowed for them to spend with their children and monitor their activities. Appropriate parent–child interactions were shaped by gender norms and by social status in the form of respectability, adolescents’ adherence to norms of respect/ obedience shaping their parents’ affection. State education affected parents’ preferences between children but also undermined parental authority, as did broader globalisation. Connectedness was related to SRH in a bi-directional way: lack of connectedness was linked to young people's low self-esteem and risky sexual behaviour while unplanned pregnancies seriously undermined young women's connectedness with their parents. Since material provision was perceived to be a central element of parent–child connectedness, structural factors limiting provision made transactional sex more likely both through direct material pathways and emotional ones. Motives for transactional sex were said to be material needs and to feel loved and cared for.

An important pathway by which structural factors shape adolescent SRH outcomes is through parent–child connectedness, especially parents’ ability to spend time with their children and provide for their economic needs. Modifying these structural factors should facilitate parent–child connectedness, which may help delay early sexual intercourse, protect young people against unplanned pregnancy through encouraging communication on contraception use and, overall, promote healthy adolescent development.

## Introduction

The World Health Organisation (WHO) defines 'connectedness' as being 'made up of behaviours that convey to adolescents that they are loved and accepted' (WHO 2007). Lay understandings of parent–child connectedness in high income countries centre on the quality of the emotional bond between parents and their children and the degree to which this bond is both mutual and sustained over time (Lezin et al. 2004). In high income countries connectedness has been shown to be protective against risky sexual behaviour resulting, for instance, in unplanned pregnancy, HIV and other sexually transmitted infections (Dittus et al. 2000, Sieving et al. 2000, Hutchinson et al. 2003, Downing et al. 2011). Other authors have also noted an association between a supportive family environment and increased condom use and confidence in negotiating condom use, and that this effect held independent of parental monitoring and parent–child communication (Crosby et al. 2002). While young people who perceive themselves to be accepted by primary caregivers are less likely to engage in a wide range of health risk behaviours, those who perceive themselves as rejected are more likely to be hostile and aggressive, have lower self-esteem and increased emotional instability (Blum 2005, Barber and Schluterman 2008).

In sub-Saharan Africa (SSA) little research has been conducted to understand the nature of parent–child connectedness. It has mainly been discussed with regards to parental physical support and communication (Kumi-Kyereme et al. 2007, Vandenhoudt et al. 2010) and there is increasing evidence from SSA of the beneficial role of parent–child communication on young people's sexual health (Mbugua 2007, Phetla et al. 2008, Poulsen et al. 2010, Vandenhoudt et al. 2010, Wamoyi et al. 2010a).

However, parent–child relationships vary across different cultures and the dimensions of these relationships probably have different meanings in different settings.

To date, sexual health promotion for young people has been largely at the level of individual behaviours, but it is increasingly recognised that risky behaviours are often driven by structural factors (Parkhurst 2012, Seeley et al. 2012), that is by underlying patterns of social systems which are beyond an individual's control. Little has, however, been done to understand the environment of young people's upbringing and the role it plays in their sexual reproduction and health (SRH). This paper explores how parents and young people in rural and peri-urban Tanzania understand the structural factors that impact on connectedness, and parents' understanding of how connectedness affects their children's sexual behaviour.

## Methods

The study took place in Magu District, Mwanza Region in northern Tanzania, amongst a rural and peri-urban population who were predominantly Sukuma. Magu was chosen because research access had previously been negotiated for other health-related topics and the research institute had previously built trust with the community. The participants were young people aged 14–24 years and parents with children within this age-group. Data were collected between July 2011 and June 2012.

Ethical approval for the study was provided by the Tanzanian Medical Research Co-ordination Committee. Additional authorisation was granted at district, ward, and village levels. In addition to seeking the consent of participants, for those aged below 18 years (the age of majority in Tanzania), consent was also sought from parents or caregivers. The purpose of, and methods for, the study were explained to potential participants, who provided verbal consent prior to participating.

### *Design*

This study employed an ethnographic research design. Data were collected using participant observation (PO), in-depth interviews (IDIs), and focus group discussions (FGDs). Combining these methods increased our understanding of complex issues related to how parents and young people interacted within their families. As noted by several authors (e.g. Murphy and Dingwall 2007), the best way to understand family interactions and the experience of family membership is by observing and interacting with families. The PO collected data on familial interactions, child socialisation and transmission of sexual norms and behaviours across generations. The FGDs focused on how participants collectively made sense of parent–child interactions, while IDIs allowed parents and young people to reflect on individual familial interactions.

### *Data generation*

Data were collected in two phases by three graduate researchers (first author included). Two were from the Sukuma ethnic group like the majority of the participants. The first phase involved PO, nine participatory FGDs and 29 IDIs, while Phase 2 involved 31 IDIs with new respondents. Furthermore, two of the young women who had had unplanned pregnancies were interviewed a second time in Phase 2 in order to pursue issues that had arisen in Phase 1.

Sampling for PO included villages, families and young people and was done with the help of the village authorities. Ten households/families were selected from one village for PO, initially on the basis of convenience sampling, recruiting people the researchers met and using their social networks to recruit others (snowball sampling). Subsequently purposive sampling was employed to ensure that the ten households/families were representative of the different household types in the community: six dual-parent households (4 non-polygamous and 2 polygamous) and four single-parent households (1 single father, 3 single mothers). The ten households were selected from about 150 households.

One of two researchers (1 male, 1 female (first author)) spent three days in each of the selected households, getting to know the families, trying to establish a trusting rapport with them, and observing the young people's familial environment. Observing naturally occurring parent-adolescent interactions was likely to provide more valid data than interview or FGD accounts, and allowed us to triangulate the IDI and FGD data. It was not intended to collect data on sexual behaviour.

A checklist was used to focus observations, including: family socio-economic status, parental presence and interactions with children, time parents spent with their children, and references to sexual relationships, young people's behaviour and unplanned pregnancies. Some of these themes were trigger points for discussions in the IDIs with some of the participants. Jottings were taken in the course of the day and detailed notes written up at the end of each day describing important observations. The two researchers met daily to review progress and their focus.

At the end of PO, FGDs and IDIs were conducted with some of the participants from the PO village and from six other villages within the same ward. As indicated in Table 1, ninety people (20 fathers, 20 mothers, 20 young women and 30 young men) participated in nine FGDs (fathers (2), mothers (2), young women (2), and young men (3). Each FGD comprised of 10 participants and conducted by a researcher of the same sex. Both purposive and snowball sampling were used for the selection of participants. With the help of the Magu demographic surveillance site field officers, initial participants (especially parents) were identified from different family types (single father, single mother, two parents) that had emerged as important for young people's upbringing during participant observation. Three days were then spent getting to know and recruiting the pre-existing friendship groups of these initial contacts, so that they knew each other well and were free to discuss sensitive issues in each other's presence (Plummer and Wight 2011). The FGDs with parents were organized according to gender, while those with young people were by gender and schooling status (in and out-of-school).

A total of 60 IDIs were conducted with: 17 fathers, 13 mothers, 13 young men and 17 (this included 2 repeat IDIs with the same participants) young women (5 of whom had had unplanned pregnancies and 10 had no children).

**Table 1:** FGD and IDI sample characteristics by research method

| Young people | | Male | | Female | |
|---|---|---|---|---|---|
| | | FGDs | IDIs | FGDs | IDIs |
| Age | 14–24 | 13 | 30 | 15[1] | 20 |
| Household type | Both parents present | 9 | 24 | 10 | 10 |
| | Single parents | 4 | 6 | 5 | 5 |
| Schooling status | Currently in school | 3 | 10 | 2 | 10 |
| | Completed primary school (currently out-of school) | 2 | 2 | 4 | 4 |
| | Some secondary school education (currently out-of school) | 7 | 7 | 8 | 8 |
| | Never been to school | 1 | 1 | 1 | 1 |
| Parents | | Male | | Female | |
| Age | 35–65 | 17 | 20 | 13 | 20 |
| Household type | Both parents present | 15 | 15 | 10 | 10 |
| | Single parents | 1 | 2 | 4 | 3 |
| Level of education | Some primary level schooling | 13 | 13 | 10 | 10 |
| | Some secondary schooling | 2 | 2 | 3 | 3 |
| | Never been to school | 2 | 2 | 0 | 0 |

[1]The total number of IDIs with young women was 17 but the number of participants interviewed was 15. This was because two of the participants were interviewed twice

In the cases of 10 of the young people interviewed, others in the same family were also interviewed: both parents (3 families), one parent (2 families) and a brother or sister (5 families). This enabled a more detailed understanding of familial interactions from the perspectives of different family members and the triangulation of responses at a family level. The remaining interviews did not involve people from the same families.

The IDIs were held with FGD participants so as to build on the rapport established during the group discussion and to explore at a personal level some of the issues that had emerged. Interviewees were purposively sampled to represent different family types, to explore experiences of unplanned pregnancy as young mothers or grandparents, and to include both dominant and reserved group discussion participants. Initially, 29 IDIs were conducted. Preliminary analysis of the Phase 1 data identified remaining gaps in our knowledge and new issues that required exploration. This was done in Phase 2 using an additional 31 IDIs with people selected through theoretical sampling (Mason 2006). The new issues explored in additional IDIs were: their views on the nature of parent–child relationships, in particular spending time together; *ukaribu* (nature of closeness/connectedness); communication; *maadili* (shared values); respect and activities that parents felt comfortable doing with their children; and the perceived SRH consequences of lack of parent–child connectedness. The responses that emerged ranged from direct discussions of the parent–child relationship to those on the environment within which parenting happens. The research was conducted in Swahili, the national language of Tanzania.

### *Analysis*

Following each of the two phases of data collection, tapes were transcribed verbatim and some translated from Swahili into English for the non-Swahili speaking senior co-investigator to confirm the emerging themes and provide feedback. All the data collected during Phase 1 and Phase 2 were entered in NVIVO 8 software for coding. A coding framework was developed in two main stages. Initially the two authors used a random selection of IDI and FGD transcripts and PO notes from Phase 1 to develop six broad codes that were used to code five FGDs and seven IDIs plus the observation notes. These codes were both a priori as well as grounded in the data and were developed in close consultation with the two other graduate fieldworkers. In the second stage the coding frame was revised in the light of Phase 2 data and to develop finer codes, again in discussion with the whole research team. All the remaining data together with that collected during Phase 2 were then coded according to the revised coding frame.

The two authors then thoroughly examined the coded data for emerging patterns which were used to formulate theories. An example of a theory was: ‘young people who reported being close to their parents were less likely to report engaging in premarital sex’. In order to test this theory, ‘child codes’ relating to spending time together, parent–child communication, parental provision of material needs, expression of parental love, feeling hopeful and optimistic about the future, and experience of unplanned pregnancy were searched. Widespread views supporting the emerging theories were examined alongside the deviant cases. In the presentation of results deviant cases are also presented as appropriate. Quotations illustrating the main findings were identified. In the presentation of the quotes, ‘PO’ refers to participant observation, ‘I’ refers to the interviewer while ‘R’ is the respondent.

## Findings

### *Socio-demographic characteristics of participants*

The participants for the study were young people aged 14–24 years (median age 20) and parents of young people within this age group. The parents were aged 35–65 years (median age 53). For most parents, the main means of livelihood was subsistence farming. A few engaged in income earning activities through petty trade within their villages and surrounding areas. The majority described themselves as Christians.

Ten of the young women who participated in IDIs lived with both parents while the remaining five lived with single mothers. For the young men, four out of thirteen lived with single mothers while the remaining nine lived with both parents. Six of the fifteen young women were unmarried mothers having had a teenage pregnancy. Five of the parents (4 mothers, 1 father) reported were single as a result of the death of their spouse.

All but four of the young people had completed primary schooling and were currently out-of-school. Most of the parents reported that they had never been to school. Five of the young people were still in school (3 boys and 2 girls). Seven of the young men and eight of the women had some secondary school education although none had been successful to continue with higher levels of secondary school. These figures are in line with the national school enrolment rates in Tanzania that indicate that 94% enrolment in primary schools and 35% enrolment in secondary school (UNICEF 2009).

### *Nature of parent–child connectedness*

When young people were asked to describe their feelings on the nature of their relationships with their parents, more than half used expressions such as, 'we live together in harmony', and 'there is no mistreatment'. Two components of positive parent–child relationships that were only mentioned by young people were the expectation of being listened to, and treated with '*upendo*' love/liking. A young man still attending school talked about what it meant to be close to one's parents:

> *'The way I see it I am close to them in a good way, ee whatever I tell them, they understand me...if I have any problem they just listen.'* [IDI, young man]

When parents were asked about how they expressed affection/love towards their children, most mentioned their main ways as being through: provision of material needs; following up on their children's activities and whereabouts; 'spending time together'; which usually referred to talking in the evening; and 'talking to their children nicely'. More than half talked about the nature of their relationships with their adolescent children using statements such as '*nampenda tu*' which means 'I just love/like her/him'. As in many languages, in both Swahili and Sukuma there are no terms to distinguish between 'love' and 'like', both being referred to by '*penda*' (Swahili). When asked what loving/liking their children meant, most fathers described this as a parent talking to a child nicely, and parental respect for their children's decisions. In a group discussion fathers described what loving/liking children meant:

> R1: *'But if you love/like the child, you talk to him nicely.'*
> R2: *'You will give him his freedom to use his right* [money]*, to use what he has earned* [cash]*...Now in that case the child will not run away from you when he sees you, he will be running towards you so that he may talk with you [father].'* [FGD, fathers]

### *Structural factors influencing parent–child connectedness*

Participants identified various influences on parent–child relationships most of which could be attributed to interrelated underlying structural factors. The structural factors with greatest influence on connectedness and ultimately child SRH outcomes were: economic circumstances, gender, system of social respectability (social status), state education and general globalisation. State education and globalisation were interlinked in that they are both a response to the changing socio-economic landscape.

### *Economic circumstances and provision*

Economic circumstances primarily affected connectedness through the material resources and time available for the children, both of which were regarded as central to parent–child connectedness. Parents' ability to provide for their children's material needs also impacted on behavioural control and parental communication. All parents talked about parent–child provision of material needs as a sign that they loved and cared for their children:

> *'Children like being given little presents, that is when they will be close to you.'* [FGD, fathers]
> *On the part of young people they perceived parental provision of their material needs as a sign of love. One said:*
> *'Father loves me more...because whatever I ask, he gives it to me...for example money for school...bus fare it is him who provides.'* [IDI, young woman]

Parents' occupations determined the time they had available to spend with their children. Parents increasingly prioritised the demands of domestic work and employment over child care as the child aged. The time mothers spent with their children declined rapidly after infancy for several reasons. Older siblings and other relatives were more likely to help care for non-breastfeeding children and from the age of seven children might go to school. Hence, the main contact that parents had with their older children was through domestic and farm activities. Some fathers acknowledged not spending sufficient time with their children:

> *'We don't have long periods maybe of saying that let us sit and talk with our children...That too can contribute in spoiling the ethics of the children.'* [FGD, fathers]

Although many young people said they were closer to their mothers than their fathers, there were a few cases of mothers spending most of their time away from home on economic activities and hence having as little time with their children as the fathers did. This was noted during PO:

> *'DK's wife is engaged in vegetable selling business and is rarely at home except for Sundays. DK said that she usually leaves home at 5am and returns at 4pm. When she arrives at home she immediately leaves to attend microcredit members' group meeting. ...DK said that he too is usually busy with church activities and their 15-year old daughter is the one responsible for most of the household chores.'* [PO notes]

Family type and marital status also determined parent time together. For example, most single parents spent little time with their children because of their income earning activities and their children were sometimes raised by other relatives including grandparents. Polygynous fathers had little time to spend with their children in any one household, given their need to distribute their time between all households.

Economic factors also shaped parent–child relationships through children's economic activities. Parents demonstrated more affection to children who contributed financially to the household and/or were hard working, and such children felt more loved, than those who did not, However, children's material contributions to the household could undermine parental authority. Parents reported that young people who made such contributions sometimes despised their parents for not providing adequately, creating barriers to parent–child connectedness.

> *'Once a child knows how to make money and, at home, the relationship with the male parent starts deteriorating.'* [IDI, father]

***Gender***

The system of gender norms had contradictory impacts on shaping parent–child connectedness. Inequitable gender relationships impacted on parent–child connectedness in several ways, especially through differential treatment of children. According to both young men and women, parents demonstrated more affection to sons than daughters, giving them more attention, requiring less domestic work from them and prioritising their education. This led young women to feel less loved by their parents, citing how their parents were stricter with them than their brothers and monitored their behaviour more.

> R1: *'A boy is loved most...Among the Sukuma traditions, actually a boy is oil.'*
> *I: 'If you say oil, what do you mean?'*
> R2: *'I mean he is just good even if he makes a mistake.'* [FGD, young women]

Gender norms stipulated appropriate parent–child interactions. Generally, both parents and young people were in agreement that whatever the degree of connectedness between children and their parents, there were limitations as to what they could discuss and who and how they would interact, primarily shaped by the social norms governing appropriate interactions across the genders. Daughters' disadvantage was slightly counter-balanced by gender norms favouring same sex communication between parents and children and mothers' greater interaction with children than fathers. A father said:

> *'It is because of their jinsia [gender]...in our Sukuma traditions it is a taboo to interfere with children of the opposite sex...that is how gender is, we don't interact across genders.'* [IDI, father]

Less than half of the fathers reported that despite sometimes feeling close to their daughters, they could only communicate with them about secretive issues (e.g. sexual health) through the mothers. They talked about the inappropriateness of their daughters communicating directly with them:

> *'A female child cannot come to ask me [father] directly...But she will go ask through her mother... so her mother is my representative in the discussion of certain things...Also for a male child, there are things he can't share with his mother directly.'* [IDI, father]

While gender norms might advantage young men in terms of resources and less domestic work, gender constraints on emotional support favour young women at the cost of young men's emotional wellbeing. If not at school, running an errand, young women were expected to be at home most of the time compared to young men. Both young men and women from two-parent families affirmed fathers' views that children feel closer to their mothers than their fathers because they spend more time with their mothers. Young women said that when they had a problem they consulted their mothers for help and then their mothers would present their problem to their father:

> *'You know many things are first channelled through mother and then she communicates with father...so you must be close to mother.'* [IDI, young woman]

While fathers acknowledged the value of being close to their children, on the other hand they talked about how being too close to one's child of either sex diminished the child's parental respect (*heshima*) due to too much familiarity. They stated the need for clear familial rules of how parents should relate with their children.

> *'I have rules as to how close I can be with my children because if you are too close, s/he will show you contempt.'* [IDI, father]

Single mothers seemed to have a bigger challenge to be close to their children than those in two parent families. All three single mothers interviewed talked of how their children, whom they had brought up out-of-wedlock, did not listen to them. They attributed this to the lack of a male adult figure in the home and their children taking them for granted because they were female and hence assumed to be less powerful.

However, although gender emerged as an important determinant of how parents interacted and expressed their love towards their children, there were a few parents and young people who felt that gender was not a barrier for them. They talked about discussing with a child or parent of the opposite sex anything they wanted. A young woman commented:

> *'We spend a lot of time sitting down with father and he gives us advice but mum, her time to sit down with us is little.'* [IDI, young woman]

***Social respectability***

Social status based on a moral system of respectability was highly salient to community members especially among fathers. Good parent–child relationships were conditional on young people's respectful behaviour, called '*heshima*'. *Heshima* was demonstrated through unquestioning obedience, correct dress code (for daughters loose clothes covering most of the body) and deference to parents, which was demonstrated especially through the '*shikamoo*' greeting, involving girls curtseying, and not initiating conversation. Almost all parents aspired that their children would demonstrate *heshima* and a child's lack of *heshima* resulted in *aibu* (shame) on one's family. This was usually due to engagement in unacceptable behaviours such as laziness or premarital sex.

Most parents reported that their children no longer followed traditions, especially those demonstrating heshima or love for their parents.

A father illustrated the role of *heshima*:

> *'Mainly it is the child, he has no love for his parent, he has no heshima...If you tell the child that you do this and that thing, he can't follow instructions; he will just do it the way he wants.'* [FGD, fathers]

Fathers also gave examples of how young people talked to their parents rudely and sometimes referred to them using slang terms:

> R1: *'He might tell you, "old man you are behind the times" [old-fashioned]...that is the language they use now'.*
>
> R2: *'Or the words 'noma, mwanangu...mzee noma huyu.'* [he is trouble, this old man is trouble].*'*
>
> *R3: 'Yeah...and many other names...you'd be called dingi* [slang for dad]*...or dingi mikwara* [dad is unnecessarily strict] *and so forth...so, you'd forbid him something but he'd say dingi mikwara.'* [FGD, fathers]

Love and *heshima* were sometimes intertwined and parents talked about loving children who had *heshima* more than those who did not. There was also the expectation of reciprocity in the relationships. Children showing *heshima* by obeying orders:

> *'You can command that "you should all go to the farm", and when they obey that order...that creates a good relationship. Therefore, when a child has a need, he will ask you and you will provide and that is a good relationship...it is because of a parent and a child helping each other.'* [IDI, father]

***State education***

State education had two contradictory impacts on parent–child connectedness. On the one hand, both parents and young people reported that parents were more affectionate and responsive to children still in school than those out-of-school, especially those who had dropped out. Children who got to secondary school, were particularly favoured. Young women were forthright in stating that their parents loved/liked them more than their siblings who were not at school. They talked about spending time with their parents, being advised to focus on their future and being provided with their material needs, including items they considered luxuries (e.g. money for hair dressing).

> *'A parent loves/likes so much the one who schools and the one who doesn't school would be isolated.'* [FGD, young men]

Conversely, many parents resented schools for undermining traditional parental authority which, they thought, led to conflict with their children. Fathers acknowledged that there were major differences in parents' and children's expectations between the present time and when they themselves grew up, when parent–child relationships were much closer. They gave an example of how state education influenced their children, undermined their traditions and child morals, and impacted on parent–child connectedness. Children who had attended secondary school were sometimes considered more disobedient and disrespectful than those who had, not partly because of feeling that they were more knowledgeable than their parents but also due to peer influence:

> *'In the past we had parental love...But now there is no love. Regardless of your good teachings, if a child leaves here* [home] *and goes to his/her peers* [at school]*, who haven't been taught well, s/he changes... while out there, s/he gets many temptations...Now in that case there won't be love.'* [FGD, fathers]

***Globalisation***

Parents reported that the nature of parent–child relationships has changed dramatically since their own childhood. They attributed these changes to both state education and more general *utandawazi* (globalisation) as reflected in mass media and modern means of communication (such as mobile telephones). They talked about how these changes have affected the way parents socialise their children. In particular, fathers blamed socio-economic changes for the deterioration of parent–child behavioural control, time together and ultimately parent–child connectedness. Reflecting on their own childhood, fathers talked about how in the past parents could sit and chat with their children at the evening fire, the *shikome*. In a conversation with a father during PO:

> *'OT* [father] *said that in the past, there were shikome, whereby young men were taught about tradition and customs about their societies and families and the rules that parents want their children to adhere to. Currently, many things have changed, there is no shikome in families and schools, video and television have spoiled everything when it comes to parenting issues. Parents have no time to spend with their children and children spend a lot of time in schools.'* [PO notes]

Parents blamed globalization as having resulted in children not following traditions especially those of respecting adults. At the broadest level globalisation was perceived as eroding traditional processes of socialisation and children increasingly saw their parents as 'living behind the times' and therefore having little to teach them as their advice was outdated. Parents talked about how it was becoming increasingly difficult for them to be close to their children because children have become more savvy [*wajanja*] than the parents:

> R1: *'Actually the children of nowadays make us parents cry, because the child doesn't listen, even if you tell him/her "do this" he doesn't want to.'*
>
> R2: *'We are struggling with them, s/he tells you "we are going with the times* [trendy]*, you old people leave us alone". Now when you are told you are old you stay feeling sad/demoralised, you ask yourself: "If you are going with the times, how come for us we grew in the old lifestyle but how come our behaviour was good, but for you who are going with the times, your behaviours are bad?"'* [FGD, mothers].

***Consequences of parent–child connectedness and young people's sexual behaviour***

Both parents and young people believed that parent–child connectedness protected 'the ethics of children' and helped prevent children from risky sexual behaviour. Reflecting on their own upbringing young women linked lack of parental love to their SRH. They mentioned: feeling isolated; engagement in premarital sex to seek emotional support/friendship from boyfriends; transactional sex since parents were not responsive to their material needs; and elopement to leave their natal homes.

### *Decision to engage in premarital sex*

It was apparent from the discussions that both parents and young people thought that the lack of parent–child connectedness would lead to risky sexual behaviour. Most of the young women reported one of the reasons for engaging in premarital sex as being due to lack of parental love and care, and hence, their desire to seek emotional support/friendship from boyfriends. A young woman acknowledged the detriments of not being close to one's parents:

> *'Not being close to one's parents is a loss. The result is having unplanned pregnancy...and sexually transmitted diseases especially HIV. You find others go beyond and start to sleep with adult men to the extent that they forget about school.'* [IDI, young woman]

Another young woman recounted her experience

> *'People were telling me, "For how long will you endure?", so I decided to join groups/peers...some were asking me, "Why do you just seat and look miserable...so, why can't you do this* [have sex]*?"…There were men who were seducing me but I had been refusing, at last I just decided to agree.'* [IDI, a young woman].

Young men shared young women's views on the consequences of poor parent–child connectedness. One gave an example of lack of parent–child connectedness leading to young women engaging in transactional sex because they did not feel close enough to express their needs:

> *'It is difficult for her to ask for money at home because she has a bad relationship with the parent. Finally, she involves herself into love affairs and gets pregnant... All this is because of having a bad relationship. If she could get money from home, she probably couldn't get it from men.'* [IDI, young man]

Most fathers acknowledged that it was important for parents, especially fathers, to spend more time with their children in order to build an atmosphere of love that would help their children feel comfortable to discuss sensitive issues, especially those related to sexual health. This was noted in PO during a discussion with a father:

> *'OE* [father] *blamed his wife for not spending time with their daughters and this resulted in their eldest daughter engaging in premarital sex. He said: "They are girls and need to be closer to their mother than me [father], but she did not spend time with them".'* [PO notes]

While young people and parents thought poor parent–child relationships contributed to young women's early sexual activity, young people also commented on how young women's sexual behaviour affected their relationships with their parents. On the positive side, some young women reported their decision to abstain from sex had enhanced their connectedness with their parents. They talked about parents preferring daughters to abstain until marriage and that parents constantly reminded them of the risks of engaging in premarital sex. A young woman talked about her behaviour of not having premarital sex as being an important contributing factor for her mother loving her:

> *'My mother loves me...even people in the streets say I am well behaved. I mean it is that respect, respect for yourself* [abstinence]*.'* [IDI, young woman]

### *Young women's experience of unplanned pregnancy*

Experiencing an unplanned pregnancy before marriage seriously undermined parent–child relationships because it led to reduced social respectability for the family and for the girl. Participants who had had an unplanned pregnancy, or had a sibling who had experienced this, said it reduced parent–daughter communication to being one-directional: parents just gave orders and warnings and the young woman was expected to obey. The only way such women could feel appreciated was when they contributed to the family finances or if they were lucky enough to receive a marriage proposal. Some contemplated suicide or eloping with any man ready to do so in order to leave their natal homes. A young woman said:

> R: *'I mean if your parent does not care for you, it causes you to think about many things. For some, they end up with many diseases...some will even elope...you get married even before you feel ready, but you just get married because they do not want you at home and whatever you do just seems bad... you will just decide on doing anything.'*
>
> I: *'And has someone ever made these decisions?'*
>
> R: *'I mean the majority do...the ones I know, they just take any men and unfortunately, get diseases... there was a girl, our neighbour, she is called "X", she became pregnant and her mother did not want her at home...she just left and got married to someone she did not know well...she now just lives a poor life.'* [IDI, young woman]

Similarly in a FGD a young woman described a mother's reaction to her daughter's pregnancy:

> *'She'd just start saying "I don't know what you've become nowadays...you'll see ... yeah, AIDS...I don't know...pregnancy...if you bear that damn baby of yours, I don't know who will take care of it for you"...so, you'd find a child [young woman] deciding even to ran away from home.'* [FGD, young women]

Although some parents whose daughters had experienced unplanned pregnancies reported spending some time with them, advising them about how to avoid further pregnancies and how to focus on their future progress, these accounts did not fit with those of most young people. All the six young women who had experienced unplanned pregnancies talked about their mothers scolding them and preferring to spend time with their siblings and not them. Two summarised their situations:

> *'I mean, I feel lonely...mum has never loved me. It is as if I do not have a mother...it is like I was not born by a mother...as in a human being, it is like... you just came like that.'* [IDI, young woman]

A second said:

> *'I mean we just live together, because there is no option, there is no love, and where else can I live?'* [IDI, young woman]

There were a few cases in which a parent's connectedness to his or her daughter increased the latter's sexual risks. For instance a few fathers used daughters as intermediaries (go-between) with their extramarital partners and some mothers encouraged their daughters' transactional sex to earn money for their families, colluding with them to conceal their sexual relationships from their fathers.

## Discussion

This paper explores the nature of parent–child connectedness, the structural factors that impact on connectedness and how connectedness affects young people's sexual behaviour in northern Tanzania. The structural factors with greatest influence on connectedness were economic circumstances, gender, social status in the form of respectability, state education and globalisation. Other authors have noted that parents' behaviour is variable and modifiable under different circumstances (Holden and Miller 1999). The factors that promote the variability or the stability in parenting practices are embedded both in family life contexts and in children's and parents' characteristics.

Our findings show that economic factors were a major barrier to parent–child connectedness as many families struggled to provide adequately for young people's material needs. Elsewhere such material contributions have been found to impact on parent–child communication (Bastien et al. 2011, Wamoyi et al. 2010a). In discussing positive parent–child relationships several parents focussed on how their material circumstances shaped these relationships. This awareness is rather different from the EuroNorth American conceptualization of positive parent–child relationships, which lays more emphasis on the emotional bond between parents and their children (Barber and Schluterman 2008, Blum 2005, Byers et al. 2003, Lezin et al. 2004, Markham et al. 2010, Miller et al. 1997, Resnick et al. 1997). Evidently economic resources are far more salient in a low income country where structural factors may play a critical role in determining the emotional climate in families, thus to investigate dimensions of parenting one also needs to investigate the conditions in which parenting occurs.

Parents blamed state education for empowering young people and disempowering parents. For example, schooling and access to modern means of communication were thought to have led young people to perceive their parents as 'old fashioned' and parents to feel ignored by their own children who perceived themselves as 'modern'. These findings confirm other studies that point to the role of socio-economic changes on parent–child relationships (Bohmer and Kirumba 2000, Goody 1989, Wamoyi et al. 2011) and point to the need for interventions to help parents cope with the emerging socio-economic circumstances. Failure to do this may lead to a backlash towards efforts that have been made to expand state education in the country as parents may be discouraged from supporting their children from pursuing such education due to fear of the consequences on social respectability.

In some cases, as with the status system of respectability, there was a circular relationship with children's more or less respectable behaviour shaping connectedness which in turn shaped children's behaviour, leading to virtuous or vicious cycles. The system of social respectability that demands children's unquestioning obedience towards adults is problematic because it undermines child autonomy and the development of individuality (Wamoyi 2008), which is an important dimension of positive parenting (WHO 2007, Barber and Schluterman 2008). Children being socialised not to question adults but to agree to their demands as a sign of respect and obedience is likely to undermine their self-esteem and hinder young women's negotiation of protection in intergenerational sexual relationships through deference to adult authority. Where intergenerational sex occurs (Silberschmidt and Rasch 2001, Luke 2003), the consequences of such a system of social respectability may need addressing. This might be approached through a parenting skills programme that shows parents that children's good behaviour can be achieved without requiring unquestioning deference to adults, and that it is important to maintain affection for one's children regardless of their behaviour.

Parents acknowledged that parent–child connectedness is enhanced by talking and listening to their children and spending time with them. If they rarely spend time together due to the nature of their economic activities as well as gender norms, it was difficult to have a close relationship with their children and to communicate effectively about sexual health. As noted elsewhere, the expression of parental love through a significant parent figure spending time with an opposite sex child was rare (Ngom et al. 2003, Babalola et al. 2005, Biddlecom et al. 2009). Our findings and those in previous studies (Twa-Twa 1997,Ngom et al. 2003) point to parental physical presence promoting positive SRH outcomes among young people.

Parents and young people were well aware of the consequences of poor parent–child connectedness on young people's SRH. They thought it led young people to: have low self-esteem; rely on peers for sexual health advice and information; and engage in risky sexual behaviour to feel loved and cared for, even if only by a sexual partner. Material provision was a core feature of feeling cared for, whether by parents or by male sexual partners. Parental poverty therefore made transactional sex more likely both through the direct material pathway of encouraging daughters to seek alternative sources of income (Wamoyi et al. 2010b, McCleary-Sills et al. 2011) and through the indirect emotional pathway by which daughters felt less connected to their parents and so sought affection elsewhere.

Conversely, young women's sexual activity also impacted on their relationship with their parents. In particular, the relationship between parents and their daughters deteriorated drastically if the daughter became pregnant unintentionally. Our findings suggest a bi-directional relationship between young women's sexual behaviour and parent–child connectedness. This can result in a vicious circle in which poor parent–child relationships prompt sexual activity which further exacerbates the parent–child relationship, especially if the daughter becomes pregnant.

This study has various limitations. First, it was conducted with a small sample in rural northern Tanzania and might not be generalisable to other locations. We acknowledge that the dynamics of parent–child interactions in urban settings may differ in many ways from rural settings. Second, while interviewing both parents and their children had many advantages, there were inconsistencies in reporting between them. While many parents including those who had had a daughter who had experienced unplanned pregnancy reported that they were close to, and loved all their children, almost all the young women who had had unplanned pregnancies indicated that they were isolated and treated badly by their parents. Such inconsistency in reporting by people from the same family may be due to social desirability bias and could indicate that parents were aware that their treatment of such daughters was undesirable. Previous research using parent–child dyads has also noted inconsistencies between parent's and children's accounts, casting doubt on their validity (Miller et al. 1998), and caution needs to be exercised when interpreting such data. This work is an initial step towards understanding parent–child connectedness and further work is required to explore how poor parent–child connectedness may lead to undesirable sexual behaviour and outcomes among young people.

Notwithstanding these shortcomings, our study contributes to understanding the context of parent–child connectedness, the structural factors impacting this and how parent–child connectedness is linked to young people's SRH risks. The findings are uniquely valuable in that they present both the perspectives of the parents and the young people themselves.

## Conclusion

Parents' and adolescents' accounts suggest that parent–child connectedness is shaped by a number of structural factors the most important being: economic circumstances; gender; respectability; state education; and globalisation. Both parents and adolescents regarded parent–child connectedness as protective against unwanted SRH outcomes, which is in line with international evidence on parent–child connectedness. These findings suggest that parenting programmes to enhance parents' understanding, skills and motivation to provide a supportive upbringing for their children, especially by being responsive to their needs and spending time with them, are unlikely to have much effect unless complemented by interventions to modify the key structural factors. A combination of these approaches should facilitate parent–child connectedness which, in turn, should help delay early sexual intercourse, protect young people against unplanned pregnancy through encouraging communication on contraception and, more generally, promote healthy adolescent development.

*Acknowledgements* – We are very thankful to all the young people and parents who participated in this study. We benefited enormously from the work of two research assistants (Mathias Shimo and Lucas Bonifas), and acknowledge their contribution. In shaping this research topic, invaluable support and advice was obtained from a colleague at London School of Hygiene and Tropical Medicine (Professor Basia Zaba) and this is highly appreciated. The undertaking to write this paper was part of a post-doctoral fellowship funded by the Medical Research Council, UK through a fellowship to the National Institute for Medical Research, Mwanza, Tanzania.
